# Theory of Charged Gels: Swelling, Elasticity, and Dynamics

**DOI:** 10.3390/gels7020049

**Published:** 2021-04-21

**Authors:** Di Jia, Murugappan Muthukumar

**Affiliations:** 1 Department of Polymer Science and Engineering, University of Massachusetts, Amherst, MA 01003, USA; djia@mail.pse.umass.edu; 2 Beijing National Laboratory for Molecular Sciences, State Key Laboratory of Polymer Physics and Chemistry, Institute of Chemistry, Chinese Academy of Sciences, Beijing 100190, China

**Keywords:** polyelectrolyte gels, elastic modulus, diffusion coefficient, dynamics, friction coefficient, mean-field theory, scaling laws

## Abstract

The fundamental attributes of charged hydrogels containing predominantly water and controllable amounts of low molar mass electrolytes are of tremendous significance in biological context and applications in healthcare. However, a rigorous theoretical formulation of gel behavior continues to be a challenge due to the presence of multiple length and time scales in the system which operate simultaneously. Furthermore, chain connectivity, the electrostatic interaction, and the hydrodynamic interaction all lead to long-range interactions. In spite of these complications, considerable progress has been achieved over the past several decades in generating theories of variable complexity. The present review presents an analytically tractable theory by accounting for correlations emerging from topological, electrostatic, and hydrodynamic interactions. Closed-form formulas are derived for charged hydrogels to describe their swelling equilibrium, elastic moduli, and the relationship between microscopic properties such as gel diffusion and macroscopic properties such as elasticity. In addition, electrostatic coupling between charged moieties and their ion clouds, which significantly modifies the elastic diffusion coefficient of gels, and various scaling laws are presented. The theoretical formulas summarized here are useful to adequately capture the essentials of the physics of charged gels and to design new hydrogels with specified elastic and dynamical properties.

## 1. Introduction

Many important hydrogels occurring in various biological contexts are constituted by charged macromolecules. Such gels also form the basis of numerous synthetically formulated soft materials with a preponderance of applications in daily life [1,2,3,4]. Yet a full theoretical description of charged hydrogels is still in progress. The difficulty in achieving a rigorous theoretical foundation resides on several key issues. First, gels are viscoelastic materials exhibiting properties of both liquid and solid states. This feature requires a theoretical formulation capable of addressing a multitude of scales of length and time. Second, the various degrees of freedom of a gel is not on equal footing, due to the unavoidable presence of slowly moving cross-links and structural inhomogeneities, compared to the polymer strands constituting the gel. This feature limits the applicability of Gibbsian statistical mechanics and requires novel tools to treat nonergodic attributes of gels. Third, there are long-range correlations among the various monomers of the gel through topological (chain connectivity), electrostatic, and hydrodynamic interactions. Fourth, since most hydrogels contain an extensive amount of water within, gels are subjected to significant levels of conformational and concentration fluctuations. Fifth, ion clouds made of small electrolyte ions always hover around the charged monomers of the gel. As a result, the dynamics of polymer strands in the gel are coupled to that of the ion cloud.

A comprehensive accounting of all of the above issues is not yet achieved. Nevertheless, considerable progress has been made to capture many of the experimentally observed phenomena on gels. Flory developed the first theory of polyelectrolyte gels [5]. In his theory, he accounted for the Donnan equilibrium, elasticity and free energy of mixing. Later, Dusek and Patterson [6], and Tanaka [7] derived equivalent forms of the Flory theory [5,8]. The electrostatic correlations and polymer conformation fluctuations are ignored in this theory. Later on, some corrections to the mean-field theory were also addressed [9,10,11]. Based on the full expression of the free energy of the charged gels, the swelling equilibrium condition, shear modulus $G_{s}$ and bulk modulus *K*, osmotic pressure Π can be derived mathematically.

Complementary to the thermodynamic treatment in the above mean field theories to address large length scales properties in equilibrium, theory of local dynamics of polymer segments were also derived. Based on the theory of Tanaka et al. [12,13]. The time evolution of the displacement vector of the gel strands is diffusion-like, and the inferred gel ‘diffusion coefficient’ $D_{g}$ is proportional to the moduli, $D_{g}=\left(K+4/3G_{s}\right)/f$, where *f* is the gel friction coefficient. This equation connects the macroscopic properties of the gels, such as elastic modulus, with microscopic properties of the gels, such as the diffusion coefficient. Besides, an analogy between swollen gels in equilibrium and semidilute polymer solutions were made by using scaling arguments [13,14]. The aforementioned theoretical approaches have been extensively discussed in attempts to explain various experimental data on many charged gels [15,16,17,18,19,20,21,22,23,24,25,26,27,28,29,30,31,32,33,34,35,36,37,38,39,40,41,42].

In this brief review, we summarize the recent generalized mean-field theory to treat polyelectrolyte gels by accounting for most of the aforementioned issues. This review is an adaption of the theory presented in an earlier publication [42]. The primary focus is the interplay between the monomeric-level microscopic dynamics and the macroscopic-level elastic properties of gels. We shall provide closed-form formulas for the state of equilibrium, elasticity, and dynamics of equilibrated gels. Furthermore, scaling laws and coupling between gel elasticity and dynamics of ion clouds will be discussed. Phase transitions and the role of nonergodic effects are not discussed in this review [9,10,13,32,33]. The theoretical predictions will be placed in the context of recent experimental findings [38,42].

The outline of this review is as follows. The theoretical model is introduced in Section 2. Derivation of free energy and the osmotic pressure of gels is given in Section 3. The equation of state of gels in terms of swelling equilibria, elasticity, and dynamics are presented in the subsequent sections, respectively, Section 4, Section 5 and Section 6. Coupling between gel dynamics and counterion dynamics is treated in Section 7. Scaling laws are presented in Section 8, followed by conclusions in Section 9.

## 2. Model Gel

Imagine a uniformly charged gel in a polar salt solution with added monovalent salt concentration $c_{s}$. Assume that there are *n* gel strands and each strand has *N* Kuhn segments. $N_{c}$ is the number of crosslinking points. We define $z_{p}$ as the number of charged groups per Kuhn segment without any counterion adsorption and α is the degree of ionization after considering counterion adsorption. Thus, the counterion concentration is $\alpha z_{p}nN/V=\alpha z_{p}c$, where *c* is the monomer number concentration (c=nN/V) and *V* is the volume of the whole gel.

**Figure 1:**
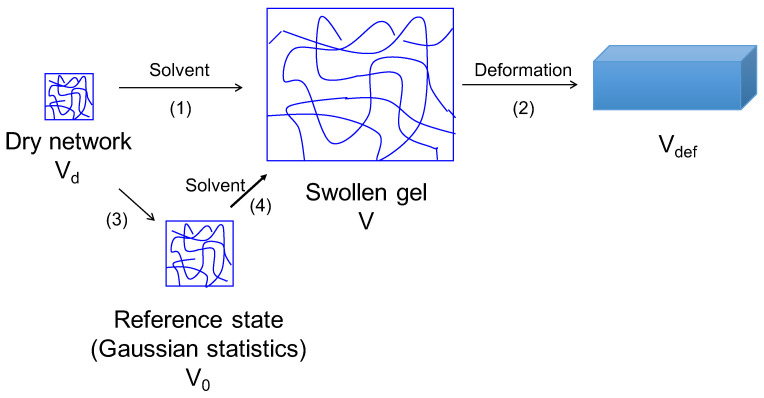
Gel behavior: (1) swelling of a gel from its dry state to its final equilibrated swollen state. (2) Deformation of an equilibrated gel due to externally imposed forces. In theoretical models, step (1) is taken as the sum of steps (3) and (4) by invoking a hypothetical reference state where the chains are assumed to obey Gaussian chain statistics.

When a gel is synthesized and purified, it has a certain initial volume. In some situations, this initial state is close to the dry state. Irrespective of the volume of the gel in the initial synthesis, let $V_{d}$ denote the volume of the gel in its dry state (Figure 1). Since the network has *n* strands each with *N* segments,

(1) $$V_{d}=nNv_{1},$$

where $v_{1}$ is the segmental volume. Note that $v_{1}$ can be chosen in different ways, such as the volume of the solvent molecule used in the Flory–Huggins theory or the cubic of Kuhn segmental length $\ell^{3}$. The specific choice of the segmental volume, accompanied by a proper adjustment of *N*, does not alter the physical conclusions but only the numerical prefactors in comparisons between theory and experiments.

Starting from the dry state of the gel, we shall discuss (1) swelling of the gel by a solvent to the equilibrated state of the gel with volume *V*, and (2) deformation of a swollen gel under tension or confinement, and gel elasticity and dynamics, as shown in Figure 1. In order to develop a molecular theory of gel behavior, it is necessary to properly account for chain statistics. Since the chain statistics in the initial state of synthesized gel is unknown and since the ideal Gaussian chain statistics is the starting point in considering the roles of all effects on chain conformations, it is convenient to invoke a hypothetical reference state for a gel where the strands between the cross-links are assumed to obey Gaussian chain statistics. The swelling process (1) is equivalent to the sum of steps (3) and (4) in Figure 1. Let the volume of the gel in the reference state be $V_{0}$. The volume fraction of the polymer in the swollen state is ϕ and that in the hypothetical state is $\phi_{0}$ given by

(2) $$\phi =\frac{V_{d}}{V}\;\;\;\;\;\;\;\;\;\;\;\;\;\;\phi_{0}=\frac{V_{d}}{V_{0}},$$

where $\phi_{0}$ is an inevitable free parameter needed to connect experiments with theoretical models.

The number of chains and the number of cross-links in the gel are related to each other. This topological relation can be quite complicated depending on the frequency of defects such as dangling ends, ring-like closed circuits, and incomplete branching emanating from the cross-link points. However, for a perfect network (without any defects), the number of *f*-functional crosslinks $N_{c}$ is related to the number of chains *n* by $N_{c}=2n/f$. When the cross-links are tetrafunctional, a perfect network of *n* chains thus has n/2 cross-links,

(3) $$N_{c}=\frac{n}{2}.$$

## 3. Full Expression for Free Energy and Derivation of Osmotic Pressure

Using Gaussian chain statistics as a reference state for the gels, the Helmholtz free energy of the gel ΔF is from the mean field part and fluctuation part [9,43,44,45]. The latter is from fluctuations in local monomer concentrations, conformations of the gel strands, and distributions of small electrolyte ions in the gel network. The net result is [9,43,44,45],

(4) $$\Delta F=\Delta F_{\mathrm{mean}\;\mathrm{field}}+\Delta F_{\mathrm{fluctuations}}.$$

The first term on the right hand side of Equation (4) arises from the free energy of mixing between the solvent and the polymers, electrostatic interactions between polymer segments, elasticity of the gels, and the Donnan equilibrium for the electrolytes. The second term is due to fluctuations. The net result is

(5) $$\Delta F_{\mathrm{mean}\;\mathrm{field}}=\Delta F_{\mathrm{mix}}+\Delta F_{\mathrm{electrostatic}}+\Delta F_{\mathrm{elastic}}+\Delta F_{\mathrm{Donnan}}.$$

These contributions are simply additive for the mean-field part. The first term on the right-hand side is the sum of contributions from the entropy of mixing between polymer and solvent, and the enthalpy due to solvent quality from the short-ranged van der Waals type of interactions. The second term is the contribution from electrostatic interactions between the charged groups mediated by mobile ions in the gel network. All kinds of contributions in Equations (4) and (5) will be detailed in the following subsections.

ΔF depends on polymer volume fraction ϕ, degree of ionization, salt concentration, the Flory-Huggins χ parameter, and cross-link density. The osmotic pressure Π and the isothermal osmotic bulk modulus *K* are given by [11,46,47]

(6) $$\Pi =-{\left(\frac{\partial \Delta F}{\partial V}\right)}_{T}=\phi^{2}\frac{\partial }{\partial \phi }{\left[\frac{\Delta F/V}{\phi }\right]}_{T};\;\;\;\;\;K=-V{\left(\frac{\partial \Pi }{\partial V}\right)}_{T}=\phi {\left(\frac{\partial \Pi }{\partial \phi }\right)}_{T}.$$

The subscript *T* indicates the isothermal condition. The various contributions to the osmotic pressure coming from the free energy due to mixing, electrostatic correlations between polymer segments, gel elasticity, Donnan equilibrium, and fluctuations are obtained from their corresponding expressions for the free energy and using Equation (6). The osmotic pressure of the gel is the sum of these contributions,

(7) $$\Pi =\Pi_{\mathrm{mix}}+\Pi_{\mathrm{electrostatic}}+\Pi_{\mathrm{elastic}}+\Pi_{\mathrm{Donnan}}+\Pi_{\mathrm{fluctuations}}.$$

When the gel is in equilibrium, the osmotic pressure of the gel must be zero,

(8) Π=0.(conditionofequilibrium)

We shall now derive expressions for the terms on the right-hand sides of Equations (4) and (7), based on simple models allowing analytical tractability. Suitable assumptions and approximations are invoked in order to track the conceptual basis for most of the major observations on gels.

### 3.1. Free Energy Due to Mixing

Assume that there are *n* chains and each chain with *N* segments. They are immersed in a solvent with total volume *V* and there are $n_{1}$ solvent molecules. The free energy of mixing is given by the Flory–Huggins theory [5] as follows:

(9) $$\frac{\Delta F_{\mathrm{mix}}}{k_{B}T}=\frac{V}{v_{1}}\left[\frac{\phi }{N}\ln\phi +\left(1-\phi \right)\ln\left(1-\phi \right)+\chi \phi \left(1-\phi \right)\right],$$

where $k_{B}T$ is the Boltzmann constant times the absolute temperature. χ is the Flory–Huggins parameter, indicating the short-ranged interactions between polymers and solvent. Here, the segmental volume equals the volume of the solvent molecule. Note that the whole gel network is regarded as a giant molecule and the center of mass is non-diffusive. Therefore, its contribution to the entropy of mixing can be ignored. As a result, the free energy of mixing is

(10) $$\frac{\Delta F_{\mathrm{mix}}}{k_{B}T}=\frac{V}{v_{1}}\left[\left(1-\phi \right)\ln\left(1-\phi \right)-\chi \phi^{2}\right],$$

where the irrelevant linear term in ϕ is ignored.

The osmotic pressure $\Pi_{\mathrm{mix}}$ corresponding to the above free energy of mixing follows from Equation (6) as

(11) $$\frac{\Pi_{\mathrm{mix}}v_{1}}{k_{B}T}=-\ln\left(1-\phi \right)-\phi -\chi \phi^{2}.$$

### 3.2. Electrostatic Energy

The electrostatic interaction energy among all segments in the gel network is given by [9,43,44,48]

(12) $$\frac{F_{\mathrm{elec}}}{k_{B}T}=\frac{1}{2}\int_{0}^{nN}ds\int_{0}^{nN}ds^{\prime }U\left[R\left(s\right)-R\left(s^{\prime }\right)\right],$$

where R(s) is the position vector of the *s*-th segment and $U\left[R\left(s\right)-R\left(s^{\prime }\right)\right]$ is the electrostatic interaction energy between the *s*-th and $s^{\prime }$-th segments. We also have

(13) $$U\left[R\left(s\right)-R\left(s^{\prime }\right)\right]=\frac{\alpha^{2}z_{p}^{2}e^{2}}{4\pi \epsilon_{0}\epsilon k_{B}T}\frac{1}{|R\left(s\right)-R\left(s^{\prime }\right)|}\exp\left[-\kappa |R\left(s\right)-R\left(s^{\prime }\right)|\right],$$

where ϵ is the dielectric constant of the medium, $\epsilon_{0}$ is the permittivity of vacuum, *e* is the electronic charge, and κ is the inverse Debye length given by

(14) $$\kappa^{2}=\frac{e^{2}}{\epsilon_{0}\epsilon k_{B}Tv_{1}}\left(\alpha z_{p}\phi +2c_{s}v_{1}\right).$$

An approximate extrapolation formula for intermediate salt concentrations can be expressed as [49]

(15) $$\frac{F_{\mathrm{elec}}}{k_{B}T}=\frac{1}{2}\frac{V}{v_{1}}\frac{\alpha^{2}z_{p}^{2}e^{2}}{\epsilon_{0}\epsilon \ell k_{B}T}\frac{{\left(nN\right)}^{2/3}}{\left[\kappa^{2}\ell^{2}{\left(nN\right)}^{2/3}+\frac{3^{4/3}\pi^{7/6}}{2^{5/3}}\phi^{2/3}\right]}\phi^{2},$$

where *ℓ* is the Kuhn segmental length and here $v_{1}$ = $\ell^{3}$. Since the volume fraction of the polymer is very small and the total number of segments is very large, $\phi^{2/3}<<\kappa^{2}\ell^{2}{\left(nN\right)}^{2/3}$, and hence $F_{\mathrm{elec}}$ becomes

(16) $$\frac{F_{\mathrm{elec}}}{k_{B}T}=\frac{1}{2}\frac{V}{v_{1}^{2}}\frac{\alpha^{2}z_{p}^{2}e^{2}}{\epsilon_{0}\epsilon \kappa^{2}k_{B}T}\phi^{2}=\frac{1}{2}\frac{V}{v_{1}}\frac{\alpha^{2}z_{p}^{2}\phi^{2}}{\left(\alpha z_{p}\phi +2c_{s}v_{1}\right)}.$$

For the two limiting cases, this reduces to

(17) $$\frac{F_{\mathrm{elec}}}{k_{B}T}=\frac{V}{v_{1}}\left\{\begin{array}{cc} \frac{1}{2}\alpha z_{p}\phi & \left(c_{s}=0\right)\\ \frac{\alpha^{2}z_{p}^{2}}{4c_{s}v_{1}}\phi^{2} & (\mathrm{high}\;\mathrm{salt}) \end{array}\right.$$

Here an effective χ parameter can be defined as [45]

(18) $$\chi_{\mathrm{eff}}=\chi -\frac{\alpha^{2}z_{p}^{2}}{4c_{s}v_{1}}.$$

Combining Equations (6), (14) and (16), and noting that $c_{s}∼V^{-1}$, the osmotic pressure $\Pi_{\mathrm{elec}}$ corresponding to the electrostatic interaction component at a given salt concentration $c_{s}$ is

(19) $$\frac{\Pi_{\mathrm{elec}}v_{1}}{k_{B}T}=0.$$

### 3.3. Free Energy Due to Deformation

Using the classical theory of rubber elasticity [5], the elastic free energy of a gel with stretching ratios $\lambda_{1},\lambda_{2},$ and $\lambda_{3}$ along the three orthogonal principal directions is given as

(20) $$\frac{F_{\mathrm{elastic}}}{k_{B}T}=\frac{n}{2}\left[\lambda_{1}^{2}+\lambda_{2}^{2}+\lambda_{3}^{2}-3-\ln\left(\lambda_{1}\lambda_{2}\lambda_{3}\right)\right],$$

where *n* is the number of strands. For isotropic swelling, where $\lambda_{1}=\lambda_{2}=\lambda_{3}=\lambda$, we can obtain

(21) $$\frac{F_{\mathrm{elastic}}}{k_{B}T}=\frac{3}{2}n\left(\lambda^{2}-1-\ln\lambda \right).$$

Noting that $V_{d}=nNv_{1},\phi_{0}=V_{d}/V_{0},\phi =V_{d}/V$, and $V=V_{0}\lambda^{3}$, we obtain

(22) $$\frac{F_{\mathrm{elastic}}}{k_{B}T}=\frac{3}{2}n\left[{\left(\frac{\phi_{0}}{\phi }\right)}^{2/3}-1-\frac{1}{3}\ln\left(\frac{\phi_{0}}{\phi }\right)\right].$$

Combining Equations (6) and (22), the osmotic pressure arising from elasticity for isotropic swelling is

(23) $$\frac{\Pi_{\mathrm{elastic}}v_{1}}{k_{B}T}=-\frac{1}{N}\left(\phi_{0}^{2/3}\phi^{1/3}-\frac{\phi }{2}\right).$$

The elastic contribution to the osmotic pressure of the gel is thus negative and works against the swelling.

Using Equations (1)–(3) and $\lambda^{3}=V/V_{0}$, $\Pi_{\mathrm{elastic}}$ can be expressed in different but equivalent forms as derived independently by Flory [5], Dusek and Patterson [6], and Tanaka [7]. For example, using $N_{c}=n/2$ and $V_{d}=nNv_{1}$, Equation (23) gives $\Pi_{\mathrm{elastic}}$ in terms of the reduced polymer concentration $\phi /\phi_{0}$ as

(24) $$\frac{\Pi_{\mathrm{elastic}}v_{1}}{k_{B}T}=-\frac{2N_{c}v_{1}}{V_{0}}\left[{\left(\frac{\phi }{\phi_{0}}\right)}^{1/3}-\frac{1}{2}\left(\frac{\phi }{\phi_{0}}\right)\right].$$

The factor $2N_{c}v_{1}/V_{0}$ is a measure of the cross-link density of the gel in the reference state, and we define it as

(25) $$\frac{2N_{c}v_{1}}{V_{0}}\equiv S\phi_{0}^{3},$$

where *S* is a parameter representing the cross-link density. In terms of *S*, Equation (24) is written as

(26) $$\frac{\Pi_{\mathrm{elastic}}v_{1}}{k_{B}T}=-S\phi_{0}^{3}\left[{\left(\frac{\phi }{\phi_{0}}\right)}^{1/3}-\frac{1}{2}\left(\frac{\phi }{\phi_{0}}\right)\right].$$

Comparing Equations (23) and (26), the cross-link density parameter *S* and the average number of segments in a chain are related by

(27) $$S\phi_{0}^{2}=\frac{1}{N}.$$

The above different, but equivalent, expressions for $\Pi_{\mathrm{elastic}}$ are intended for the reader to follow different notations used by different investigators on gel theory.

The above formulas are based on the effective chains being sufficiently long and the net stretching forces sufficiently low to warrant the applicability of Gaussian statistics used in arriving at Equation (20). However, if the strands are short enough such that the root mean square end-to-end distance of an effective chain is comparable to its contour length, the finite extensibility of the chain must be included [5].

### 3.4. Osmotic Pressure from Mobile Ions and Donnan Equilibrium

The electrolyte ions will exchange inside and outside of the gel network until the gel reaches equilibrium when the interior and exterior of the gel network are each charge neutral and the chemical potential of the electrolytes is uniform. Such an equilibrium is defined as Donnan equilibrium. Let the electrolyte ions be monovalent. The osmotic pressure of the gel due to the equilibrated electrolytes, $\Pi_{\mathrm{ion}}$, is given under ideal conditions as [5]

(28) $$\frac{\Pi_{\mathrm{ion}}v_{1}}{k_{B}T}=\sqrt{\alpha^{2}z_{p}^{2}\phi^{2}+4v_{1}^{2}c_{s}^{2}}-2v_{1}c_{s}.$$

### 3.5. Fluctuations

An analogy between a semidilute solution above the overlap concentration and a swollen gel can be drawn such that the free energy of the system per correlation volume $\xi^{3}$ is the thermal energy $k_{B}T$, where ξ is the correlation length for monomer concentration fluctuations arising from chain connectivity. Based on the double screening theory of polyelectrolyte systems [9,43], the free energy due to conformational fluctuations is given as

(29) $$\frac{\Delta F_{\mathrm{fluc}}}{k_{B}T}∼\frac{V}{\xi^{3}},$$

where ξ is self-consistently related to the Debye screening length $\kappa^{-1}$. As well known [9,43], $\xi ∼\phi^{-3/4}$ in the high salt limit and $\xi ∼\phi^{-1/2}$ in the salt-free limit. Furthermore, based on the Debye–Hückel theory [50], the free energy contribution from the electrostatic correlations of electrolyes is given by

(30) $$\frac{\Delta F_{\mathrm{DH}}}{k_{B}T}=-\left(\frac{V}{v_{1}}\right)\frac{1}{4\pi }\left[\ln\left(1+\kappa \ell \right)-\kappa \ell +\frac{1}{2}\kappa^{2}\ell^{2}\right].$$

In addition, fluctuations in local polymer concentrations also play a role for the free energy of the gel, especially when the gel is near critical points.

In the derivation of swelling equilibrium discussed in the next subsection, we mainly focus on the mean-field theory without considering fluctuations.

## 4. Swelling Equilibrium of Isotropically Swollen Gels

The total osmotic pressure of the gel from all of the above mentioned contributions (Equations (11), (19), (23) and (28)) is given as

(31) $$\frac{\Pi v_{1}}{k_{B}T}=-\ln\left(1-\phi \right)-\phi -\chi \phi^{2}+\sqrt{\alpha^{2}z_{p}^{2}\phi^{2}+4c_{s}^{2}v_{1}^{2}}-2c_{s}v_{1}-\frac{1}{N}\left(\phi_{0}^{2/3}\phi^{1/3}-\frac{\phi }{2}\right).$$

**Figure 2:**
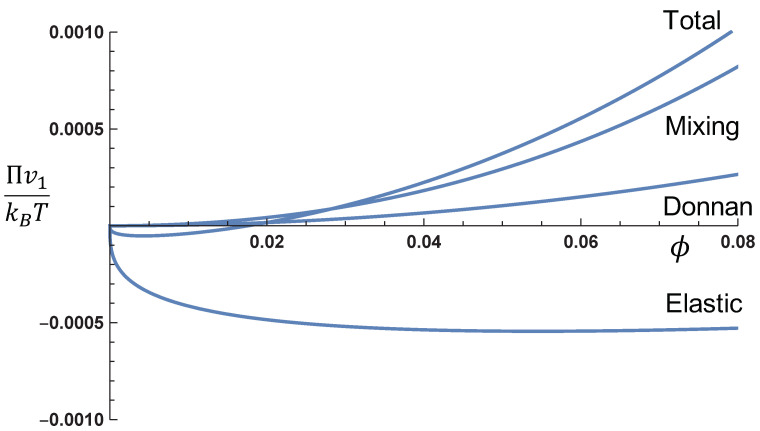
Contributions of mixing, Donnan, and elastic terms to the total osmotic pressure $\Pi v_{1}/k_{B}T$ at $c_{s}=0.1$ M. ($\chi =0.4,\alpha =0.1,z_{p}=1,v_{1}=1\;{\mathrm{nm}}^{3},\phi_{0}=0.1$, and N=100.)

**Figure 3:**
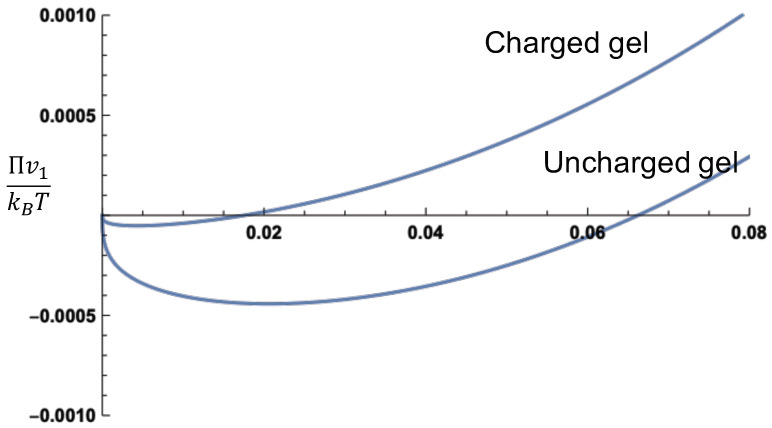
Comparison of the osmotic pressures of an uncharged gel and a charged gel. The values of the parameters are the same as in Figure 2.

In order to gain insight into the relative values of the mixing, Donnan, and elastic contributions, Figure 2 gives these contributions and the total osmotic pressure ($\Pi v_{1}/k_{B}T$) as functions of the polymer volume fraction at the monovalent salt concentration $c_{s}=0.1$ M. Note that the values of $\chi ,\alpha ,z_{p},v_{1},N$, and $\phi_{0}$ are system dependent. As an example of the behavior of the three contributions to the osmotic pressure, we have taken χ=0.4, α=0.1, $z_{p}=1,v_{1}=1\;{\mathrm{nm}}^{3},\phi_{0}=0.1$, and N=100. The osmotic pressure from the mixing contribution and the Donnan contribution is both positive, with the mixing term stronger than the Donnan contribution for a particular choice of the parameters used in this figure. On the other hand, the elastic contribution leads to negative osmotic pressure in the polymer concentration presented in the figure. As a compromise between these opposing pressures, equilibrium is attained at Π=0. In Figure 2, the swelling equilibrium occurs at ϕ=0.0188. A comparison between an uncharged gel and a charged gel is given in Figure 3, where the values of the various parameters are the same as in Figure 2. It is obvious from this figure that the osmotic pressure of a charged gel is higher than that of an uncharged gel. This is entirely due to the Donnan contribution.

At the swelling equilibrium, Π=0, so that the condition of swelling equilibrium of charged gels follows from Equation (31) as

(32) $$-\ln\left(1-\phi \right)-\phi -\chi \phi^{2}=-\sqrt{\alpha^{2}z_{p}^{2}\phi^{2}+4c_{s}^{2}v_{1}^{2}}+2c_{s}v_{1}+\frac{1}{N}\left(\phi_{0}^{2/3}\phi^{1/3}-\frac{\phi }{2}\right).$$

The above equation is the generalization of the Flory–Rehner theory [8] of swelling equilibrium of neutral gels to polyelectrolyte gels. We now proceed to derive the limiting behaviors of swelling equilibrium in the salt-free limit and high salt limit.

### 4.1. Salt-Free Gels

When $c_{s}=0$, the swelling equilibrium for isotropically swollen gels follows from Equation (32) as

(33) $$-\ln\left(1-\phi \right)-\phi -\chi \phi^{2}+\alpha z_{p}\phi =\frac{1}{N}\left(\phi_{0}^{2/3}\phi^{1/3}-\frac{\phi }{2}\right).$$

Expanding the logarithmic term for ϕ<<1, and for $\alpha z_{p}>>1/2N$, we get

(34) $$\phi^{2/3}=\frac{\phi_{0}^{2/3}}{\alpha z_{p}N}.$$

The ratio of the gel volume to the volume of the dry network, called the swelling ratio, follows as

(35) $$\frac{V}{V_{d}}=\frac{1}{\phi }=\frac{{\left(\alpha z_{p}N\right)}^{3/2}}{\phi_{0}}.$$

Here $\phi_{0}$ is the parameter defined in Equation (2) due to the necessity of invoking a reference state for the gel with Gaussian chain statistics. The swelling ratio of the volume of the gel is proportional to the 3/2 power of the effective number of counterions per chain in the gel. In the salt-free strong swelling regime, the swelling ratio is independent of the χ parameter.

**Figure 4:**
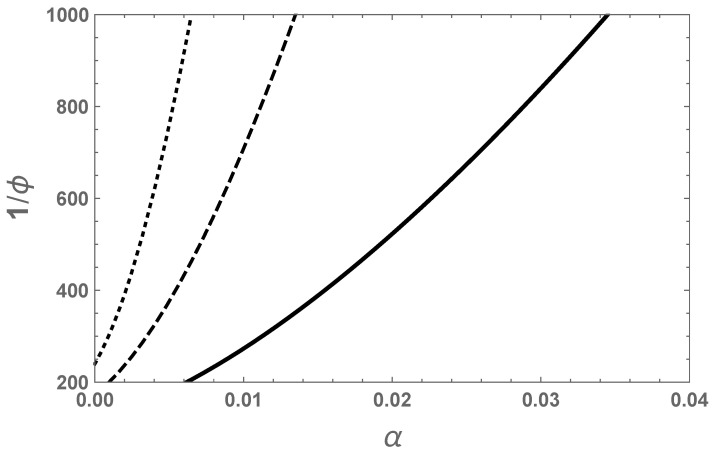
Dependence of the swelling ratio on degree of ionization and chain length for $c_{s}=0$, χ=0, $\phi_{0}=0.008$, and *N* = 100 (solid), 250 (dashed), and 500 (dotted).

The dependence of the swelling ratio on the degree of ionization as given by Equation (33) for $c_{s}=0$ is given in Figure 4. As the average number of segments per chain increases, the swelling ratio increases. The curvature of the traces in Figure 4 is due to the 3/2 power law given in Equation (35). As seen from the figure, the swelling ratio can be substantial even for a very small degree of ionization. For such strong swelling, the assumed Gaussian chain statistics cannot be adequate and the inverse Langevin function must be used to correctly predict the swelling equilibrium.

### 4.2. Gels with High Salt

For $c_{s}v_{1}>>\alpha z_{p}\phi$, Equation (32) gives

(36) $$-\ln\left(1-\phi \right)-\phi -\chi_{\mathrm{eff}}\phi^{2}+0\left(\phi^{3}\right)=\frac{1}{N}\left(\phi_{0}^{2/3}\phi^{1/3}-\frac{\phi }{2}\right)$$

where

(37) $$\chi_{\mathrm{eff}}=\chi -\frac{1}{4}\frac{\alpha^{2}z_{p}^{2}}{c_{s}v_{1}}.$$

Note that $\chi_{\mathrm{eff}}$ is of the same form given in Equation (18). The modification of χ by inter-segment electrostatic interactions does not contribute to the osmotic pressure as given in Equation (19). However, χ is modified into $\chi_{\mathrm{eff}}$ due to the additional contribution from mobile electrolytes under Donnan equilibrium. Thus at higher salt concentrations, the contribution from the mobile ions to the properties of the gel is negligible due to electrostatic screening and alleviation of Donnan pressure. As a result, the effective χ is essentially the same as the χ value for the polymer–solvent combination which usually leads to phase separation. When $c_{s}$ is decreased, $\chi_{eff}$ becomes more negative, making the background fluid of the gel a better solvent. If the bare χ is sufficiently positive, so that the uncharged gel is in a poor solvent and thus can collapse, charging the gel will stabilize the swollen gel with fewer strong electrolyte ions. However, upon the addition of more salt, the $\chi_{eff}$ approaches the bare χ leading to the collapse of the gel.

For small values of polymer concentration, ϕ<<1, which is typical for swollen gels, the logarithmic term of Equation (36) can be expanded to give the swelling equilibrium as

(38) $$\phi^{5/3}≃\frac{\phi_{0}^{2/3}}{N\left(\frac{1}{2}-\chi_{\mathrm{eff}}\right)}.$$

Therefore, the swelling ratio for the gel in the limit of high salt is

(39) $$\frac{1}{\phi }≃{\left(\frac{N(\frac{1}{2}-\chi_{\mathrm{eff}})}{\phi_{0}^{2/3}}\right)}^{3/5}.$$

**Figure 5:**
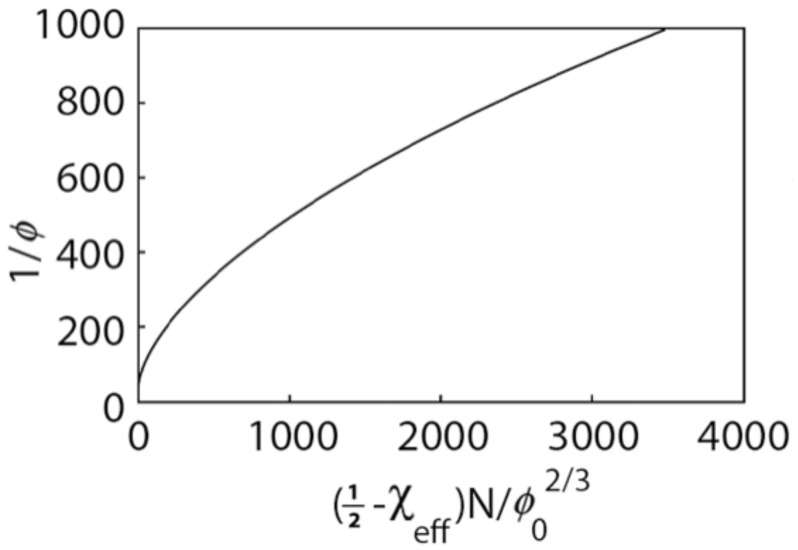
Dependence of the swelling ratio on chain length in the high-salt limit. ($\chi_{\mathrm{eff}}=0,\phi_{0}=0.008$).

Thus the swelling ratio of the gel in the high salt limit is proportional to the 3/5 power of the average number *N* of segments in the elastically active strands between cross-links. As *N* increases (that is, as the crosslink density decreases), the swelling ratio increases. The dependence of the swelling ratio on $(\frac{1}{2}-\chi_{\mathrm{eff}})N$ is plotted in Figure 5 for $\chi_{\mathrm{eff}}=0$ and $\phi_{0}=0.008$. As the chain length increases, the swelling ratio is higher. For large values of $(\frac{1}{2}-\chi_{\mathrm{eff}})N$, the behavior is in accordance with the 3/5 power law given in Equation (39).

**Figure 6:**
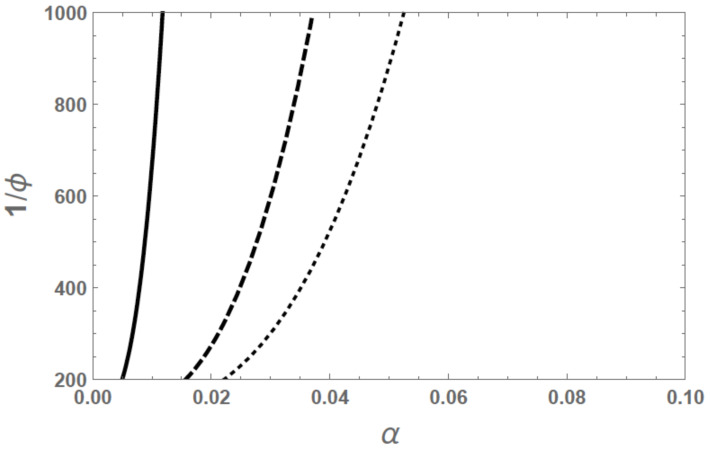
Dependence of the swelling ratio on degree of ionization and intermediate salt concentrations for $\chi =0,\phi_{0}=0.008$, and N=100. $c_{s}v_{1}=0.001$ (solid), 0.01 (dashed), and 0.02 (dotted).

The above results are the generalization of the Flory–Rehner theory [8] to polyelectrolyte gels, where $\chi_{\mathrm{eff}}$ is simply χ. The crossover behavior between Equations (35) and (39) at intermediate salt concentrations is given by the full formula of Equation (32). A typical result is illustrated in Figure 6, where the swelling ratio is plotted against the degree of ionization for different salt concentrations. As the salt concentration is reduced, the swelling ratio increases drastically for a fixed degree of ionization. The swelling ratio is higher at a higher degree of ionization.

## 5. Elasticity of Swollen Gels

Consider the situation where a swollen gel is pulled out of the liquid and then subjected to deformation by keeping the polymer volume fraction fixed. The various elastic properties of equilibrated gels depend on polyelectrolyte concentration, degree of ionization, pH, and salt concentration. By considering simple elongation and simple shear, we shall present stress-strain relations and molecular expressions for Young’s modulus, shear modulus, and bulk modulus of equilibrated swollen gels.

### 5.1. Simple Elongation

Consider a simple uniaxial stretching of a swollen gel, where the stretching is along the longitudinal direction 1, and the two transverse directions, 2 and 3, are equivalent. Let $\lambda_{s}=L_{1}/L$ be the stretching ratio with respect to the unstretched swollen gel, and $\lambda_{1}=L_{1}/L_{0}$ be the stretching ratio with respect to the reference state (due to both swelling and stretching). From Equation (20) and $L/L_{0}={\left(\phi_{0}/\phi \right)}^{1/3}$ (Equation (2)), we get

(40) $$\lambda_{1}=\frac{L_{1}}{L_{0}}=\frac{L_{1}}{L}\frac{L}{L_{0}}=\lambda_{s}{\left(\frac{\phi_{0}}{\phi }\right)}^{1/3}.$$

Now $\phi_{0}/\phi$ is a constant and does not depend on deformation. Assuming that there is no volume change during the stretching of the swollen gel,

(41) $$\lambda_{1}\lambda_{2}\lambda_{3}=\frac{V}{V_{0}}=\frac{\phi_{0}}{\phi }.$$

Therefore,

(42) $$\lambda_{2}=\lambda_{3}=\sqrt{\frac{}{}}\phi_{0}\lambda_{1}\phi =\frac{1}{{\sqrt{\lambda }}_{s}}{\left(\frac{\phi_{0}}{\phi }\right)}^{1/3}.$$

Hence, from Equation (20), we get the free energy change due to elastic deformation as

(43) $$\Delta F_{elastic}=\frac{nk_{B}T}{2}\left[\lambda_{s}^{2}{\left(\frac{\phi_{0}}{\phi }\right)}^{2/3}+\frac{2}{\lambda_{s}}{\left(\frac{\phi_{0}}{\phi }\right)}^{2/3}-3-\ln\left(\frac{\phi_{0}}{\phi }\right)\right].$$

The tensile stress $\sigma_{t}$ is given by

(44) $$\sigma_{t}=\frac{1}{L^{2}}\frac{\partial }{\partial L_{1}}\Delta F_{elastic}=\frac{1}{L^{3}}\frac{\partial }{\partial \lambda_{s}}\Delta F_{elastic}.$$

Combining Equations (43) and (44), we get

(45) $$\sigma_{t}=\frac{nk_{B}T}{V}{\left(\frac{\phi_{0}}{\phi }\right)}^{2/3}\left(\lambda_{s}-\frac{1}{\lambda_{s}^{2}}\right).$$

Since $\phi =V_{d}/V$ and $V_{d}=nNv_{1}$,

(46) $$\sigma_{t}=\frac{k_{B}T}{Nv_{1}}\phi_{0}^{2/3}\phi^{1/3}\left(\lambda_{s}-\frac{1}{\lambda_{s}^{2}}\right).$$

The Young’s modulus *E* follows from this equation as

(47) $$E={\left(\frac{\partial \sigma_{t}}{\partial \lambda_{s}}\right)}_{\lambda_{s}\to 1}=\frac{3k_{B}T}{Nv_{1}}\phi_{0}^{2/3}\phi^{1/3}.$$

### 5.2. Simple Shear

As well known, for an incompressible system, the shear strain γ is related to the deformation $\lambda_{s}$ as

(48) $$\gamma =\lambda_{s}-\frac{1}{\lambda_{s}},$$

where the deformation along the principal axes are [51,52]

(49) $$L_{1}=\lambda_{s}L,\;\;\;\;\;\;\;\;\;\;L_{2}=\frac{1}{\lambda_{s}}L,\;\;\;\;\;\;\;\;\;\;L_{3}=L.$$

Therefore,

(50) $$\lambda_{1}=\frac{L_{1}}{L_{0}}=\frac{L_{1}}{L}\frac{L}{L_{0}}=\lambda_{s}{\left(\frac{\phi_{0}}{\phi }\right)}^{1/3};\;\;\lambda_{2}=\frac{1}{\lambda_{s}}{\left(\frac{\phi_{0}}{\phi }\right)}^{1/3};\;\;\lambda_{3}={\left(\frac{\phi_{0}}{\phi }\right)}^{1/3}.$$

Note that $\lambda_{1}\lambda_{2}\lambda_{3}=\phi_{0}/\phi =V/V_{0}$, as expected.

The free energy change due to simple shear follows from Equations (20) and (50) as

(51) $$\Delta F_{elastic}=\frac{nk_{B}T}{2}\left[\lambda_{s}^{2}{\left(\frac{\phi_{0}}{\phi }\right)}^{2/3}+\frac{1}{\lambda_{s}^{2}}{\left(\frac{\phi_{0}}{\phi }\right)}^{2/3}+{\left(\frac{\phi_{0}}{\phi }\right)}^{2/3}-3-\ln\left(\frac{\phi_{0}}{\phi }\right)\right].$$

Using Equations (48) and (51) is rewritten in terms of the strain γ as

(52) $$\Delta F_{elastic}=\frac{nk_{B}T}{2}{\left(\frac{\phi_{0}}{\phi }\right)}^{2/3}\gamma^{2}+\mathrm{terms}\;\mathrm{in}\;(\phi_{0}/\phi \;),$$

where γ is defined in Equation (48).

The shear stress $\sigma_{s}$, defined as $\sigma_{s}=\partial \left(\Delta F_{elastic}/V\right)/\partial \gamma$, follows from Equation (52) as

(53) $$\sigma_{s}=\frac{nk_{B}T}{V}{\left(\frac{\phi_{0}}{\phi }\right)}^{2/3}\gamma .$$

Thus, the shear modulus $G_{s}$ is

(54) $$G_{s}=\frac{nk_{B}T}{V}{\left(\frac{\phi_{0}}{\phi }\right)}^{2/3}.$$

In view of $V=V_{d}/\phi =nNv_{1}/\phi$, we get

(55) $$G_{s}=\frac{k_{B}T}{Nv_{1}}\phi_{0}^{2/3}\phi^{1/3}.$$

Comparing Equations (46) and (55), the tensile stress can be written in terms of shear modulus as

(56) $$\sigma_{t}=G_{s}\left(\lambda_{s}-\frac{1}{\lambda_{s}^{2}}\right).$$

The expression for the shear modulus given by Equation (55), derived for incompressible gels, is a general expression that does not require equilibrium. The validity of Equation (55) has been validated experimentally by measuring the shear modulus of nearly ideal gels with fixed architecture [25]. On the other hand, if different gels of unknown structure are prepared, then the prefactor representing the structure (proportional to 1/N) is not known. However, the factor containing *N* in Equation (55) can be eliminated [38,42] using the swelling equilibrium condition given by Equation (32). As s result, the shear modulus is given as

(57) $$\frac{G_{s}v_{1}}{k_{B}T}=-\ln\left(1-\phi \right)-\phi -\chi \phi^{2}+\sqrt{\alpha^{2}z_{p}^{2}\phi^{2}+4c_{s}^{2}v_{1}^{2}}-2c_{s}v_{1}+\frac{\phi }{2N}.$$

For ϕ<<1 and for *N* so large that ϕ/(2N) is negligible, the shear moduli in the two limiting cases follow as

(58) $$\frac{G_{s}v_{1}}{k_{B}T}=\left\{\begin{array}{cc} \left(\alpha z_{p}\right)\phi & \left(c_{s}=0\right)\\ \left(\frac{1}{2}-\chi +\frac{\alpha^{2}z_{p}^{2}}{4c_{s}v_{1}}\right)\phi^{2} & (\mathrm{high}\;\mathrm{salt}) \end{array}\right.$$

Note that ϕ is determined at the swelling equilibrium condition, since the derived shear modulus is applicable for equilibrated gels.

For the low-salt limit, $G_{s}$ is directly proportional to the polymer concentration, while for the high-salt limit, $G_{s}$ is proportional to the square of the polymer concentration. The predicted quadratic dependence, $G_{s}∼\phi^{2}$, is observed [38] in experiments on weakly cross-linked hyaluronic acid hydrogels with concentrations of added NaCl ranging from 10${}^{-3}$ M to 1 M. This quadratic scaling behavior is also observed [42] in poly(acrylamide-co-acrylate) hydrogels with 10% charge density containing 0.005, 0.01, 0.1, and 1.0 M NaCl. In these experiments, the different polymer concentrations of the swollen gels are obtained by choosing different values of cross-link density and allowing the gels to attain their ultimate equilibrium, while keeping the salt concentration fixed. The quadratic dependence $G_{s}∼\phi^{2}$ derived in the high salt limit is observed even at the salt concentration of 0.005 M NaCl, indicating that even for such low salt concentrations, theoretical expressions derived for the high salt limit may be applicable.

The slope of the plot of $G_{s}v_{1}/k_{B}T$ versus $\phi^{2}$ is given by Equation (58) as

(59) $$\frac{\partial (G_{s}v_{1}/k_{B}T)}{\partial \phi^{2}}=\frac{1}{2}-\chi +\frac{\alpha^{2}}{4c_{s}v_{1}}.$$

The last term arises from the inter-segment electrostatic interactions and the Donnan equilibrium. A plot of the above slope against $1/c_{s}$ allows an experimental method to determine the value of χ and the effective degree of ionization in charged gels.

In general, Equation (58) can be used to relate a macroscopic elastic property such as shear modulus to molecular characteristics such as degree of ionization and strand length. Equation (58) provides design rules for tuning the shear modulus of hydrogels.

### 5.3. Osmotic Bulk Modulus

The bulk modulus *K* of isotropically swollen gels follows from Equations (6) and (31) as

(60) $$\frac{Kv_{1}}{k_{B}T}=\frac{\phi }{1-\phi }-\phi -2\chi \phi^{2}+\sqrt{\alpha^{2}z_{p}^{2}\phi^{2}+4c_{s}^{2}v_{1}^{2}}-2c_{s}v_{1}-\frac{1}{3N}\left(\phi_{0}^{2/3}\phi^{1/3}-\frac{3}{2}\phi \right).$$

This expression reduces to simple laws for the concentration dependence of the osmotic bulk modulus of swollen gels for salt-free and high salt limits as given below.

**(i)**  **Salt-free limit**:

For $c_{s}=0$ and ϕ<<1, the above equation simplifies to

(61) $$\frac{Kv_{1}}{k_{B}T}≃\left(\alpha z_{p}+\frac{1}{2N}\right)\phi -\frac{1}{3N}\phi_{0}^{2/3}\phi^{1/3},$$

where terms of order $\phi^{2}$ are ignored. Combining with Equation (33), we get

(62) $$\frac{Kv_{1}}{k_{B}T}=\frac{2}{3}\left(\alpha z_{p}+\frac{1}{2N}\right)\phi .$$

For $\alpha z_{p}>>1/2N$, this result becomes

(63) $$\frac{Kv_{1}}{k_{B}T}≃\frac{2}{3}\alpha z_{p}\phi .$$

Therefore, according to the mean-field theory used in deriving the above results, the osmotic bulk modulus in the salt-free situation is proportional to αϕ, just as the shear modulus. In addition, the prediction from such a simple theory is that the osmotic bulk modulus is 2/3 of the shear modulus in this limit.

**(ii)**  **High salt limit**:

For $2c_{s}v_{1}>>\alpha z_{p}\phi$ and ϕ<<1, Equation (60) becomes

(64) $$\frac{Kv_{1}}{k_{B}T}=\left(1-2\chi +\frac{\alpha^{2}z_{p}^{2}}{4c_{s}v_{1}}\right)\phi^{2}-\frac{1}{3N}\phi_{0}^{2/3}\phi^{1/3}.$$

Combining Equations (38) and (64), we get

(65) $$\frac{Kv_{1}}{k_{B}T}=\frac{5}{3}\left(\frac{1}{2}-\chi +\frac{1}{10}\frac{\alpha^{2}z_{p}^{2}}{c_{s}v_{1}}\right)\phi^{2}.$$

Using Equation (37), the osmotic bulk modulus can be alternatively written as

(66) $$\frac{Kv_{1}}{k_{B}T}=\frac{5}{3}\left(\frac{1}{2}-\chi_{\mathrm{eff}}-\frac{3}{20}\frac{\alpha^{2}z_{p}^{2}}{c_{s}v_{1}}\right)\phi^{2}≃\frac{5}{3}\left(\frac{1}{2}-\chi_{\mathrm{eff}}\right)\phi^{2}.$$

In view of the derived expression for the shear modulus $G_{s}$, Equation (58), the relation between the bulk modulus and the shear modulus in the high salt limit follows from Equation (66) as

(67) $$K≃\frac{5}{3}G_{s}.$$

Note that both *K* and $G_{s}$ are proportional to $\phi^{2}$ at high salt concentrations and in equilibrium, according to the mean-field theory presented above.

## 6. Dynamics of Charged Gels

In the gel network, each segment fluctuates around its equilibrium position r, so the displacement vector u of a polymer segment in the gel is $u=r^{\prime }-r$. Based on the theory of elasticity [12], The equation of motion for u is given as

(68) $$f\frac{\partial u}{\partial t}=G_{s}\nabla^{2}u+\left(K+\frac{1}{3}G_{s}\right)\nabla \left(\nabla \cdot u\right).$$

Here, inertia is ignored. *f* is the friction coefficient of the polymer network against the solvent per unit volume. Taking the volume element as the mesh of the gel with average linear size ξ, the gel friction coefficient per unit volume can be written as

(69) $$f=\frac{6\pi \eta_{0}\xi }{\left(\frac{4}{3}\pi \xi^{3}\right)}=\frac{9\eta_{0}}{2\xi^{2}},$$

where $\eta_{0}$ is the viscosity of background fluid,

For the longitudinal mode, u propagates along the longitudinal direction *x*, ∇(∇·u) in Equation (68) reduces to $\nabla^{2}u$ so that [12]

(70) $$\frac{\partial u_{\ell }}{\partial t}=\left(\frac{K+\frac{4}{3}G_{s}}{f}\right)\frac{\partial^{2}u_{\ell }}{\partial x^{2}},$$

where $u_{\ell }$ is the longitudinal component of the displacement vector u. Equation (70) is identical to the well-known diffusion equation. Therefore, we define the prefactor on the right hand side as gel diffusion coefficient [15]

(71) $$D_{g}=\frac{K+\frac{4}{3}G_{s}}{f}\equiv \frac{M_{\ell }}{f},$$

where $M_{\ell }$ is the longitudinal modulus defined as $M_{\ell }=K+\frac{4}{3}G_{s}$. Another explanation for $D_{g}$ is the cooperative diffusion coefficient, which is related to an effective correlation length of concentration fluctuations in the gel network, analogous to that in semidilute solutions [13],

(72) $$D_{g}=\frac{k_{B}T}{6\pi \eta_{0}\xi }.$$

The effective correlation length in the above equation originates from dynamics accounting for screened hydrodynamics, electrostatic interactions, and screened excluded volume. On the other hand, the correlation length obtained from static light scattering measurements does not include contributions from hydrodynamic correlations and their coupling with excluded volume and electrostatic interactions. However, these two screening lengths are proportional to each other, as well known in the literature [46,53,54].

The gel diffusion coefficient can be measured by dynamic light scattering, and the correlation length can be obtained from static light scattering measurements. Experiments on aqueous poly(acrylamide-co-acrylate) gels containing 0.01 M NaCl show [42] that $D_{g}$ is proportional to $\phi^{2/3}$ and ξ is proportional to $\phi^{-2/3}$, in agreement with the prediction of Equation (72). In order to make connection between the microscopic quantity $D_{g}$ with the macroscopic quantity related to the elastic moduli, as given in Equation (71), it is necessary to measure the friction coefficient *f*. Based on water permeation measurements, *f* is found to be proportional to $\phi^{4/3}$ for the same poly(acrylamide-co-acrylate) gels where $D_{g}∼\phi^{2/3}$ and $G_{s}∼\phi^{2}$ are observed [42]. Furthermore, since $G_{s}∼K∼\phi^{2}$ (Equations (58) and (65)) in the high-salt limit, the experimentally observed results $f∼\phi^{4/3}$ and $D_{g}∼\phi^{2/3}$ are internally self-consistent with Equation (71).

## 7. Coupling between Gel Dynamics and Counterion Dynamics

Analogous to the dynamics of polyelectrolyte solutions in the context of the ‘ordinary–extraordinary’ transition [40,54,55,56], where the counterion cloud is generally coupled to the segmental dynamics, the segmental dynamics of charged gels is coupled to the dynamics of the counterion cloud surrounding the segments. Generalizing the equations [54,55] for polyelectrolyte solutions to gels, where the cooperative diffusion coefficient in solutions is replaced by the gel diffusion coefficient, we get the the following coupled equations for salt-free gels,

(73) $$\frac{\partial \delta c_{1}}{\partial t}=-\left(\frac{M_{\ell }}{f}\right)k^{2}\delta c_{1}-\left(\frac{c_{1}^{0}}{f}\right)\frac{\alpha z_{p}e^{2}c_{1}^{0}}{\epsilon_{0}\epsilon }\left(\alpha z_{p}\delta c_{1}+z_{c}\delta c_{2}\right)$$

(74) $$\frac{\partial \delta c_{2}}{\partial t}=-D_{2}k^{2}\delta c_{2}-D_{2}\frac{z_{c}e^{2}c_{2}^{0}}{\epsilon_{0}\epsilon k_{B}T}\left(\alpha z_{p}\delta c_{1}+z_{c}\delta c_{2}\right).$$

Here, $\delta c_{1}$ is the fluctuation in the local polymer concentration from its average value $c_{1}^{0}$ and $\delta c_{2}$ is the fluctuation in the local counterion concentration from its average value $c_{2}^{0}$. $D_{2}$ is the cooperative diffusion coefficient of the counterion without any coupling to the polymer matrix. $z_{p}$ and $z_{c}$ are the valencies of the segment and counterion, respectively, α is the degree of ionization, *e* is the electronic charge, $\epsilon_{0}$ is the permittivity of vacuum, and ϵ is the dielectric constant of the gel medium. In the presence of added salt, additional equations similar to Equation (74) appear for each electrolyte ionic species in the gel [40,57].

The first term on the right-hand side of Equation (73) is due to the diffusive flux given in Equation (70) and the second term is due to the electrostatic coupling between the charged segments and their counterion clouds. Following the same procedure as for polyelectrolyte solutions, and assuming that the counterion clouds relax much faster than the gel, the rate of change of fluctuation in local polymer concentration is given by

(75) $$\frac{\partial \delta c_{1}\left(k,t\right)}{\partial t}=-D_{g,\mathrm{coupled}}k^{2}\delta c_{1}\left(k,t\right),$$

where

(76) $$D_{g,\mathrm{coupled}}=\frac{M_{\ell }}{f}+\frac{\alpha^{2}z_{p}^{2}e^{2}{\left(c_{1}^{0}\right)}^{2}}{f\epsilon_{0}\epsilon \kappa^{2}},$$

where κ is the inverse Debye length. For monovalent salt ions and $z_{p}=1$, $\kappa^{2}$ is given by $\kappa^{2}=4\pi \ell_{B}\left(\alpha c_{1}^{0}+2c_{s}\right)$, with $\ell_{B}$ being the Bjerrum length.

Therefore, the coupling between the ion cloud and gel leads to an additional contribution to the enhancement of $D_{g}$ with decreased salt concentration and increased degree of ionization. In any quantitative comparison with experiments, Equation (76) needs to be employed.

## 8. Scaling Laws

So far in this chapter, we have presented only the mean-field theory of charged gels without accounting for concentration and conformational fluctuations in the system. The advantage of the above derivations is the ability to get closed-form analytical formulas enabling direct comparison with the numerical values of experimental data. Although fluctuations are ignored above, the derived results are in qualitative agreement with experimental findings as mentioned above. Nevertheless, it is useful to extract scaling laws, such as power-law dependence of the elastic moduli on polymer concentration, from the above equations. Before doing this, let us revisit the role of fluctuations as introduced in Equations (4) and (29).

The contribution to the free energy from concentration fluctuations can be written using scaling arguments as

(77) $$\Delta F_{\mathrm{fluctuations}}≃k_{B}T\frac{V}{\xi^{3}},$$

where ξ is the correlation length for monomer concentration fluctuations. This is basically equivalent to the argument [46] that the energy of the system per correlation volume is the thermal energy $k_{B}T$. If the fluctuations dominate the free energy of the gel over the mean field contribution, the scaling laws for the osmotic pressure and osmotic modulus are

(78) $$\Pi ∼\frac{T}{\xi^{3}},$$

(79) $$K=c\frac{\partial \Pi }{\partial c}∼\frac{T}{\xi^{3}}.$$

As we have already noted in Equation (69), the scaling law for the gel friction coefficient is

(80) $$f∼\frac{\eta_{0}}{\xi^{2}}.$$

Assuming that $G_{s}$ is proportional to bulk modulus *K*, we get the scaling law for the gel diffusion coefficient as

(81) $$D_{g}=\frac{M_{\ell }}{f}∼\frac{T}{\eta_{0}\xi }.$$

This result is internally self-consistent with the generalized Stokes-Einstein law for the cooperative diffusion coefficient given by Equation (72).

A common assumption in the literature is that the scaling behavior in swollen gels containing salt is the same as the scaling behavior in semidilute solutions under similar salt conditions [13,14,21,46]. For sufficiently high salt concentrations, the polyelectrolyte solutions behave as good solutions and hence the correlation length obeys the scaling law $\xi ∼c^{-3/4}$ by using the size exponent ν=3/5 for good solutions in the scaling law, $\xi ∼c^{-\nu /(3\nu -1)}$. This would imply the following scaling laws for swollen gels with high salt,

(82) $$\xi ∼c^{-3/4},\;\;\;\;\;K∼c^{9/4},\;\;\;\;\;f∼c^{3/2},\;\;\;\;\;\mathrm{and}\;\;\;\;\;D_{g}∼c^{3/4}.$$

However, systematic experiments on weakly cross-linked hyaluronic acid gels and aqueous poly(acrylamide-co-acrylate) gels containing salt show the following scaling laws [38,42]

(83) $$\xi ∼c^{-2/3},\;\;\;\;\;K∼c^{2},\;\;\;\;\;f∼c^{4/3},\;\;\;\;\;\mathrm{and}\;\;\;\;\;D_{g}∼c^{2/3},$$

where $\xi ,G_{s},f$, and $D_{g}$ are determined independently using static light scattering, rheology, water permeation, and dynamic light scattering, respectively, for equilibrated swollen gels [38,42]. These laws are precisely the results already mentioned, predicted by the mean-field theory described in this chapter, and are internally self-consistent by satisfying Equations (80)–(82). Note that this self-consistency among the scaling laws is observed, although fluctuations are completely ignored in the theory.

Since $\xi ∼c^{-\nu /(3\nu -1)}$ under semidilute conditions, and $\xi ∼c^{-2/3}$ in Equation (84), the effective size exponent for the weakly cross-linked gels investigated in the above experiments is 2/3 and not the value of 3/5 nominally used in good solutions. With the value of 2/3 for ν, the scaling law for the dependence of the correlation length on $\chi ,\alpha ,c_{s}$, and *c* is given by

(84) $$\xi ∼{\left(\frac{1}{2}-\chi +\frac{\alpha^{2}z_{p}^{2}}{4c_{s}v_{1}}\right)}^{-1/3}c^{-2/3}.$$

Substituting this result in Equation (72), the dependence of the elastic diffusion coefficient of gels on $\chi ,c_{s},\alpha$, and *c* is given by

(85) $$D_{g}∼\frac{T}{\eta_{0}}{\left(\frac{1}{2}-\chi +\frac{\alpha^{2}z_{p}^{2}}{4c_{s}v_{1}}\right)}^{1/3}c^{2/3}.$$

The predictions of the above equation are borne out to be valid as seen in experiments [15,42].

The demonstrated consistency of predictions of the mean-field theory with experiments provides confidence in using the derived equations as design principles to tune the desired elastic properties of charged hydrogels. Deviations are expected if the contribution from fluctuations to the free energy of the gel dominates over the mean-field component, and if structural inhomogeneities inside the gel and charge regularization are significant.

## 9. Conclusions

A mean-field theory has been developed for polyelectrolyte gels by accounting for the free energy of mixing, elasticity, electrostatic interactions among segments and the Donnan equilibrium. Moreover, osmotic pressure, osmotic bulk modulus, shear modulus, gel friction coefficient, and gel diffusion coefficient are expressed as closed-form equations. Furthermore, we have addressed the modification of gel diffusion coefficient by the coupling of charged segments with their ion clouds from counterions and electrolyte ions.

The corroborating experimental results also support the following scaling laws,

(86) $$\xi ∼\phi^{-2/3},\;\;\;\;\;K∼\phi^{2},\;\;\;\;\;f∼\phi^{4/3},\;\;\;\;\;\mathrm{and}\;\;\;\;\;D_{g}∼\phi^{2/3}.$$

If conformational fluctuations play a significant role, the above scaling laws will be modified.

A key summary of this review is that the mean-field theory works remarkably well for such a complicated charged gel system, without considering conformational fluctuations. The distinction between the results outlined here and results in the early literature on gels can be traced to the preparation of the experimental system. In earlier investigations, different parameters such as degree of ionization, salt concentration, and polymer concentration are coupled, instead of varying only one variable by keeping the other variables fixed.

Yet, the fluctuation part can dominate over the mean filed part when the gel reaches the critical boundaries for phase transition, which is not addressed here.

## Data Availability

The details of derivations presented here are available on request from the corresponding author.
